# Dynamic Dimerization of Chemokine Receptors and Potential Inhibitory Role of Their Truncated Isoforms Revealed through Combinatorial Prediction

**DOI:** 10.3390/ijms242216266

**Published:** 2023-11-13

**Authors:** Mengke Li, Rui Qing, Fei Tao, Ping Xu, Shuguang Zhang

**Affiliations:** 1 Laboratory of Molecular Architecture, Media Lab, Massachusetts Institute of Technology, Cambridge, MA 02139, USA; lmk328@mit.edu; 2 State Key Laboratory of Microbial Metabolism, Joint International Research Laboratory of Metabolic and Developmental Sciences, School of Life Sciences and Biotechnology, Shanghai Jiao Tong University, Shanghai 200240, China; ruiqing.br@sjtu.edu.cn (R.Q.); taofei@sjtu.edu.cn (F.T.); pingxu@sjtu.edu.cn (P.X.)

**Keywords:** AlphaFold2 prediction, complex structure prediction, GPCR oligomerization, molecular docking, transmembrane truncation

## Abstract

Chemokine receptors play crucial roles in fundamental biological processes. Their malfunction may result in many diseases, including cancer, autoimmune diseases, and HIV. The oligomerization of chemokine receptors holds significant functional implications that directly affect their signaling patterns and pharmacological responses. However, the oligomerization patterns of many chemokine receptors remain poorly understood. Furthermore, several chemokine receptors have highly truncated isoforms whose functional role is not yet clear. Here, we computationally show homo- and heterodimerization patterns of four human chemokine receptors, namely CXCR2, CXCR7, CCR2, and CCR7, along with their interaction patterns with their respective truncated isoforms. By combining the neural network-based AlphaFold2 and physics-based protein–protein docking tool ClusPro, we predicted 15 groups of complex structures and assessed the binding affinities in the context of atomistic molecular dynamics simulations. Our results are in agreement with previous experimental observations and support the dynamic and diverse nature of chemokine receptor dimerization, suggesting possible patterns of higher-order oligomerization. Additionally, we uncover the strong potential of truncated isoforms to block homo- and heterodimerization of chemokine receptors, also in a dynamic manner. Our study provides insights into the dimerization patterns of chemokine receptors and the functional significance of their truncated isoforms.

## 1. Introduction

There are 27 chemokine receptors that are members of the class A G protein-coupled receptors (GPCRs) [1]. They initiate cellular signaling pathways upon binding with extracellular signaling molecules, including 36 chemokines, thereby regulating a myriad of biological activities. These receptors play pivotal roles in various physiological processes, particularly immune surveillance, inflammation, neurotransmission, and cell migration [2]. The dysregulation of chemokine receptors contributes to the initiation or progression of a wide spectrum of diseases, including autoimmune diseases, cancer, heart disease, and HIV [3]. Consequently, chemokine receptors have been regarded as prime targets for drug development. Up to now, many drugs targeting chemokine receptors have been successfully developed and applied in clinical therapeutics [4], including Maraviroc, the HIV entry inhibitor targeting CCR5 [5], and Mozobil, the stem cell mobilizer targeting CXCR4 [6].

The oligomerization of GPCRs has been extensively demonstrated to hold significant functional implications [7,8], and it is known to be essential for the cell surface delivery of many class A and class C GPCRs [9]. In recent years, an increasing body of research has indicated the impact of chemokine receptor homo/hetero-oligomerization on their functionality [3,10,11]. Generally, oligomerization alters ligand binding performance and signaling pathways of chemokine receptors, thereby affecting diverse physiological activities, including cell migration [12,13], viral infection [14], embryonic development [15], and cancer progression [16]. However, there still remains a lack of studies on GPCR oligomerization patterns at the structural level. The challenges can be summarized as follows: (i) For structure determination, GPCRs, as membrane proteins, are challenging to prepare in substantial quantities and crystallize [17]; (ii) the dynamic nature of GPCR oligomerization [7,18] makes it difficult to draw unambiguous conclusions about its patterns; and (iii) GPCR oligomerization is influenced by various factors, including (a) ligand concentrations, (b) ligand types [19,20], (c) the composition [21,22], and (d) geometry [23] of the membrane environment, and (e) protein density [23]. Therefore, numerous computational studies have emerged that predict GPCR oligomerization patterns or study the molecular dynamics using available oligomer structures [24]. Many of them support the dynamic nature of GPCR oligomerization [22,25,26]. It is worth noting that the revolutionary protein structure prediction tool AlphaFold2 [27] has demonstrated remarkable capabilities in predicting complex structures [28,29,30,31], holding the potential to significantly advance structural research on protein complexes.

Protein isoforms, derived from tissue-specific alternative splicing, represent an important source of functional diversity [32]. Some GPCR isoforms exhibit distinct signaling properties [33,34,35]. A systematic study by Marti-Solano et al. [36] summarized topological types of human GPCR isoforms and their functional characteristics and revealed distinct combinatorial expression patterns across different tissues. Interestingly, some GPCR isoforms are significantly truncated, no longer possessing the seven-pass transmembrane feature; nevertheless, they display unique functional roles [37]. Some can interfere with cell surface trafficking and/or reduce the ligand binding affinity of full-length counterparts by dimerizing with them [38,39,40,41,42], while some even show well-preserved functionality [43,44].

However, the mechanism through which these truncated isoforms interact with their full-length counterparts and whether chemokine receptors also possess these functional features remain largely unexplored. We serendipitously discovered that a truncated chemokine receptor variant, CCR5-SZ190b, can individually perform signaling, albeit at a reduced level [45]. We found that many human CC and CXC chemokine receptors have truncated isoforms deposited in the UniProt database, and we compiled their data (Supplementary Table S1 and Figure S1). The majority of them are also deposited in the GPCR isoform database established by Marti-Solano et al. [36], and their tissue-specific expression profiles have been well documented.

We here report the computational study of the homo- and heterodimerization patterns of four chemokine receptors, CXCR2, CXCR7, CCR2, and CCR7, as well as their interaction patterns with their respective truncated isoforms. By combining the neural network-based AlphaFold2 and the physics-based protein–protein docking tool ClusPro [46], we predicted 15 groups of complex structures and yielded a total of 90 models. We assessed the binding affinities of the complexes using the Molecular Mechanics Poisson–Boltzmann Surface Area (MMPBSA) algorithm [47] in the context of atomistic molecular dynamics simulations. Our results of the chemokine receptor homo- and heterodimerization interfaces and binding intensity show considerable consistency with some previous experimental observations and support the dynamic and diverse nature of GPCR dimerization patterns. Furthermore, we predicted the presence and patterns of their interactions with their respective truncation isoforms, strongly suggesting the potential inhibitory role of these truncated isoforms in the dimerization of full-length receptors.

## 2. Results

### 2.1. Workflow of Interface Prediction and Analysis

**Figure 1:**
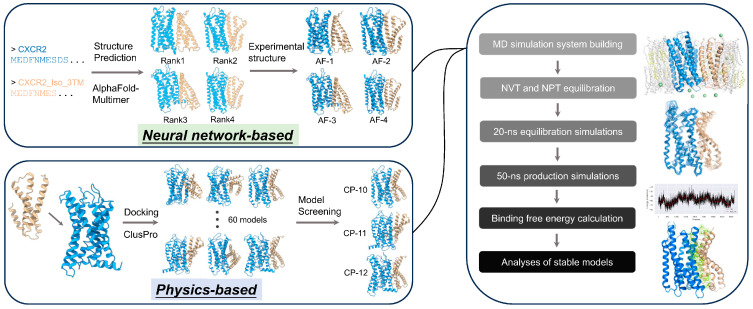
The workflow of our computational approach. The workflow of our computational approach. Briefly, we generated initial complex models by combining the neural network-based protein structure prediction tool AlphaFold2 and the physics-based protein–protein docking tool ClusPro. For complex structure prediction, we employed AlphaFold-Multimer to generate five models, from which the models with unique interface compositions were selected. These models were then aligned with experimental structures to replace predicted structures (except isoforms). For protein–protein docking, ClusPro generated 60 models, from which topology-compliant models were chosen. Both sets of initial complex models were used as inputs to build protein complex–membrane systems for MD simulations. After NVT (constant number of atoms, volume, and temperature) and NPT (constant number of atoms, pressure, and temperature) equilibration, a 20 ns equilibration MD simulation was run to achieve the stabilization of the complexes. This was followed by a 50 ns production MD simulation, subsequently subjected to binding free energy calculations based on the MMPBSA algorithm. According to the results, the most stable complex models were selected for further analysis. The structural snapshots were taken using PyMOL or ChimeraX.

We selected the chemokine receptors whose experimental structures are available and whose truncated isoforms are deposited in the UniProt database (Supplementary Table S1), namely CXCR2, CXCR7, CCR2, and CCR7, as the subjects of our study. Complex models were generated using two distinct methodologies (Figure 1). We employed AlphaFold-Multimer [28] to build five models, from which those with unique interface compositions were selected, followed by aligning them with experimental structures and replacing the predicted structures (except isoforms). In parallel, we generated 60 models using ClusPro and chose those models that were topology-compliant. The topology-compliant models were defined when the following rules were met: (i) The orientation of both protomers was consistent with a typical GPCR orientation; (ii) the putative membrane layers inferred from transmembrane regions of both protomers were basically spatially consistent; and (iii) if it was a homodimer model, the interface composition of both protomers was identical. Next, we used these initial complex models to construct protein–membrane (containing cholesterol) systems and performed all-atom molecular dynamics (MD) simulations. The post-equilibration 50 ns simulation trajectories were subjected to binding free energy (BFE) calculations using the MMPBSA algorithm [47]. After comparing BFE, the most stable (i.e., with the lowest BFE) models were selected for further analysis. It is worth noting that for those complexes in which two or three models were stable, or they were comparably stable, and their interfaces on the subject protomer were distinct, we analyzed all stable models, considering the diversity and dynamics of GPCR interaction modes, along with computational errors.

The results of all complex models are summarized in Supplementary Tables S2–S5. For each complex, four to eight models were input into MD simulations and BFE calculations. We designated the truncated isoforms as ‘Iso_XTM’ to highlight the number of remaining transmembrane helices and the extent of truncation; for example, “Iso_2TM” denotes the truncated isoform with two transmembrane helices. We found that the most stable models (Table 1) could originate from both AlphaFold and ClusPro, although ClusPro models dominated, likely due to the physics-based nature of ClusPro. We observed significant diversity in interface composition, with each transmembrane helix potentially serving as the interface core, except for the conservatively highly buried TM3. Surprisingly, most truncated isoforms exhibited notably strong binding strengths, binding to the surfaces overlapping with the dimerization interfaces of chemokine receptors. In the following sections, we will present a detailed analysis of each subject chemokine receptor.

### 2.2. CXCR2: Blocking Capability of Three Different Truncated Isoforms

**Figure 2:**
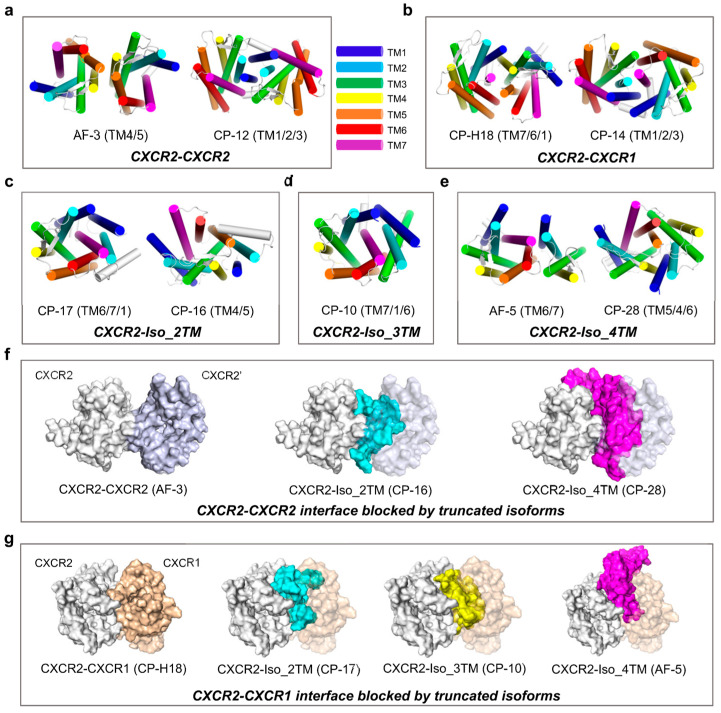
Interaction patterns of CXCR2 homodimers, heterodimers, and complexes with its truncated isoforms: (**a**–**e**) Top view of the conformations of the most stable complex models of CXCR2-CXCR2 (**a**), CXCR2-CXCR1 (**b**), CXCR2-Iso_2TM (**c**), CXCR2-Iso_3TM (**d**), and CXCR2-Iso_4TM (**e**), identified through binding free energy calculations. Two models displayed in one box indicate that both of the two models are stable or they are comparably stable, and their interfaces on CXCR2 are distinct. For every model, the designation and the information of the interface on CXCR2 are shown below the structure. Regarding the interface composition, the order of the helices is consistent with Table 1, i.e., it is determined by their contribution to the interface formation. The transmembrane helices are colored according to the scheme shown on the right side of (**a**). Other parts of the structures are shown in white. (**f**,**g**) CXCR2-CXCR2 (**f**) and CXCR2-CXCR1 (**g**) interfaces are blocked by truncated isoforms, shown in the top view. The structures are shown as surfaces. To show the blocking effect, we aligned the subject protein structure (here, it is CXCR2) in the dimer model with that in the subject-blocker model, followed by hiding the subject protein structure in the subject-blocker model. All structural snapshots were taken from the medoid frame in the largest conformational cluster using PyMOL.

CXCR2 has been confirmed to constitutively form homodimers [19,48,49] and heterodimers with CXCR1 [19,49]. Our results showed two homodimer models with distinct interfaces but with comparable binding strengths: model AF-3 with the TM4/TM5 interface and model CP-12 with the TM1/TM2/TM3 interface (Figure 2a). It is worth noting that this study mainly focuses on the interface of the subject protein. Specifically, in this section, the TM4/TM5 interface refers to the interface composed of TM4 and TM5 of CXCR2. Similarly, two CXCR2-CXCR1 heterodimer models exhibit distinct interfaces with close binding intensity: model CP-H18 with the TM7/TM6/TM1 interface and model CP-14 with the TM1/TM2/TM3 interface (Figure 2b). We observed that the TM1/TM2/TM3 interface is shared by both the homodimer and heterodimer, sterically almost completely overlapping when aligning the models (Supplementary Figure S2). This implies that CXCR2 and CXCR1 could compete for dimerization with CXCR2 at the TM1/TM2/TM3 interface, and the binding strength of the heterodimer (BFE = −110.32 ± 0.23 kcal/mol) exceeds that of the homodimer (−69.82 ± 0.33 kcal/mol), consistent with the previous observation indicating that CXCR1 expression can significantly decrease the population of CXCR2-CXCR2 homodimers [19]. To enable a better understanding of the BFE value calculated using MMPBSA, we calculated the BFE of the CXCR4 homodimeric structure (PDB: 3ODU) under the same parameters, which was −23.18 ± 0.14 kcal/mol.

We identified three distinct truncated isoforms of CXCR2 in the UniProt database, designated according to the number of transmembrane helices, namely CXCR2_Iso_2TM (henceforth abbreviated as Iso_2TM), Iso_3TM, and Iso_4TM (Supplementary Table S1). These isoforms possess complete N-termini but exhibit varying degrees of truncation at the C-termini (Supplementary Figure S1a). Both Iso_2TM and Iso_4TM display two stable models with different interfaces, while Iso_3TM shows a single stable model (Figure 2c–e). We found that both Iso_2TM (BFE = −104.69 ± 0.29 kcal/mol) and Iso_4TM (−96.96 ± 0.20 kcal/mol) could block CXCR2 homodimerization (−70.52 ± 0.14 kcal/mol) at the TM4/TM5 interface, with the binding areas closely overlapping (Figure 2f). The blocker was considered to have the ability to block the dimerization interface when the following rules were met: (i) The interface on the subject in the dimer model had a significantly overlapped portion with the interface on the subject in the subject-blocker model; (ii) the binding free energy of the subject-blocker model was lower than or close to that of the dimer model. Furthermore, we found that Iso_2TM (BFE = −133.80 ± 0.18 kcal/mol), Iso_3TM (−123.76 ± 0.22 kcal/mol), and Iso_4TM (−105.62 ± 0.19 kcal/mol) could all block CXCR2-CXCR1 heterodimerization (−118.83 ± 0.19 kcal/mol) at the TM6/TM7 interface (Figure 2g).

**Figure 3:**
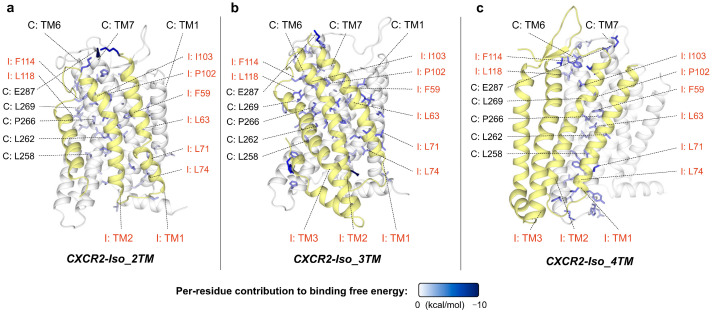
The three different truncated isoforms of CXCR2 interact with CXCR2 in a similar manner: (**a**–**c**) Side view of the interfaces of CXCR2-Iso_2TM (**a**), CXCR2-Iso_3TM (**b**), and CXCR2-Iso_4TM (**c**). The residues that positively contribute (per-residue contribution < −1 kcal/mol) to interface formation are shown as sticks, and in blue, with the color intensity determined by their contribution to binding free energy (as the bar shown below the diagram). CXCR2 backbones are shown in white and the isoform backbones are shown in yellow. For the labels, “C” denotes CXCR2, and “I” denotes the isoform. The labeled residues are those significantly contributing to all three CXCR2–isoform interfaces. The labels related to CXCR2 are shown in black with a dashed connector, and those related to isoforms are shown in orange. Here, we only show the single most stable model for each CXCR2–isoform complex. All structural snapshots were taken from the medoid frame in the largest conformational cluster using PyMOL.

We found that these truncated isoforms are not simply products of structural removal of other components. They interact with full-length CXCR2 not by merely removing other portions from the homodimer structure; instead, they interact with distinct conformations to achieve greater binding strength than the homodimer. We observed that either side of the truncated isoforms, exposed or buried when aligned to full-length CXCR2, could comprise the interface (Supplementary Figure S3a,b), showing comparable binding strength. Overall, the three truncated isoforms maintain consistency in binding strength (Table 1) and interaction pattern (Figure 3). The most stable single model of each CXCR2–isoform complex shows the interface is mainly composed of TM6/TM7 of CXCR2 and TM1/TM2 of the isoform. We found a set of residues shared by all three CXCR2–isoform models that significantly contributed to the interface formation (Figure 3). These residues are spread uniformly throughout the interface in the three models. This consistency supports the existence of their interaction with full-length CXCR2 and the reliability of our computational methodology. Nonetheless, we can still observe some differences in their interaction patterns, and basically, the three truncated isoforms exhibit a modest reduction in binding strength with increasing length, i.e., Iso_2TM > Iso_3TM > Iso_4TM. The relatively less extensive interface also mirrors the weaker binding intensity of Iso_4TM (Figure 3c). We speculate that this could be explained by the conformational constraints and structural flexibility, which could also account for their competence to block native dimerization with higher binding strength.

### 2.3. CXCR7: Highly Stable CXCR7-CXCR7 and CXCR7-CXCR4, and Competitive Dimerization

**Figure 4:**
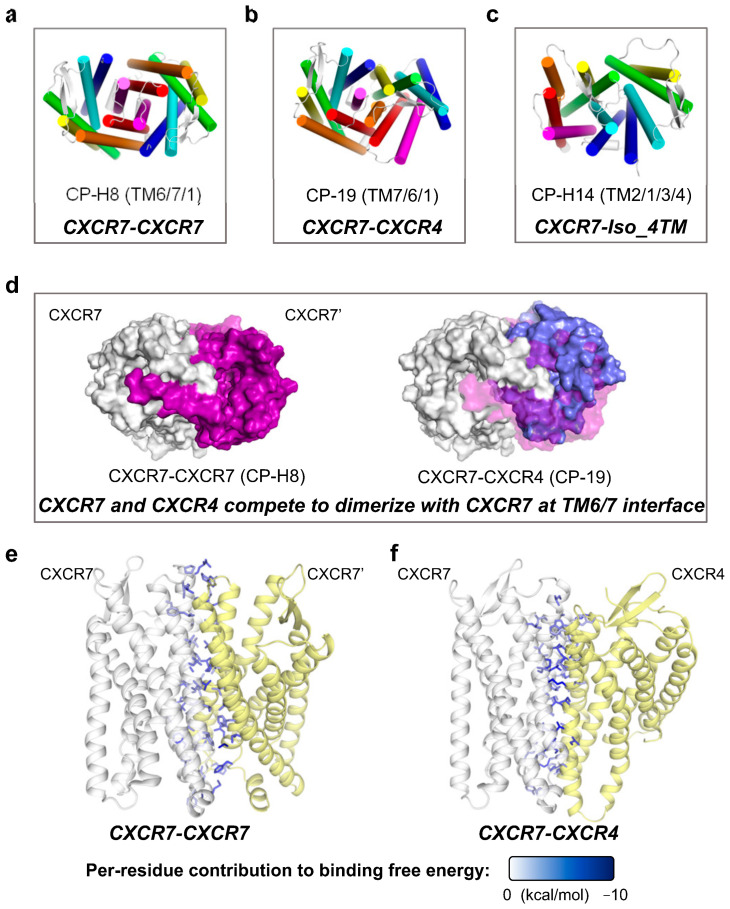
Interaction patterns of CXCR7 homodimers, heterodimers, and complexes with its truncated isoforms: (**a**–**c**) Top view of the conformations of the most stable complex models of CXCR7-CXCR7 (**a**), CXCR7-CXCR4 (**b**), and CXCR7-Iso_4TM (**c**), identified by binding free energy calculations. The display style is the same as that in Figure 2a. (**d**) CXCR7 and CXCR4 compete for dimerization with CXCR7 at TM6/TM7 interface. The display style is the same as that in Figure 2f. (**e**,**f**) Side view of the interfaces of CXCR7-CXCR7 homodimer (**e**) and CXCR7-CXCR4 heterodimer (**f**). The display style is the same as that in Figure 3. All structural snapshots were taken from the medoid frame in the largest conformational cluster using PyMOL.

CXCR7 (also known as ACKR3) can form constitutive homodimers [50,51,52] and heterodimers with CXCR4 [15,51,52]. From our results (Figure 4a,b), both CXCR7 homodimer and CXCR7-CXCR4 heterodimer show an exceptionally stable model with binding strength far higher than other models within the same complex group (Supplementary Table S3). The interfaces of these two models exhibit a high degree of overlap, and their binding strengths are closely aligned (homodimer: −160.12 ± 0.41 kcal/mol; heterodimer: −150.17 ± 0.21 kcal/mol), indicating competition between CXCR7 and CXCR4 for dimerization with CXCR7 at the TM6/TM7 interface (Figure 4d). Previous studies indicated that CXCR7-CXCR4 heterodimerization is as efficient as CXCR7 homodimerization [52] and the co-expression of these two receptors triggers a stronger Ca^2+^ flux than CXCR4 alone [15], which is in line with our results. These two models represent the highest binding strengths among all chemokine receptor dimer models in this study, evident from the tight spacing between the interface helices from their respective protomers (Figure 4a,b) and the abundance of the residues highly contributing to interface formation (Figure 4e,f). The notable stability of both homodimer and heterodimer suggests that CXCR7 might inherently favor dimer formation.

CXCR7 has a truncated isoform with four transmembrane helices (4TM), similarly truncated at the C-terminus (Supplementary Figure S1b). However, this truncated isoform does not exhibit the potential to block dimerization due to its moderate binding strength and the lack of interface overlap in the CXCR7-Iso_4TM model.

### 2.4. CCR2: Competitive Dimerization and Blocking Capability of the Truncated Isoform

**Figure 5:**
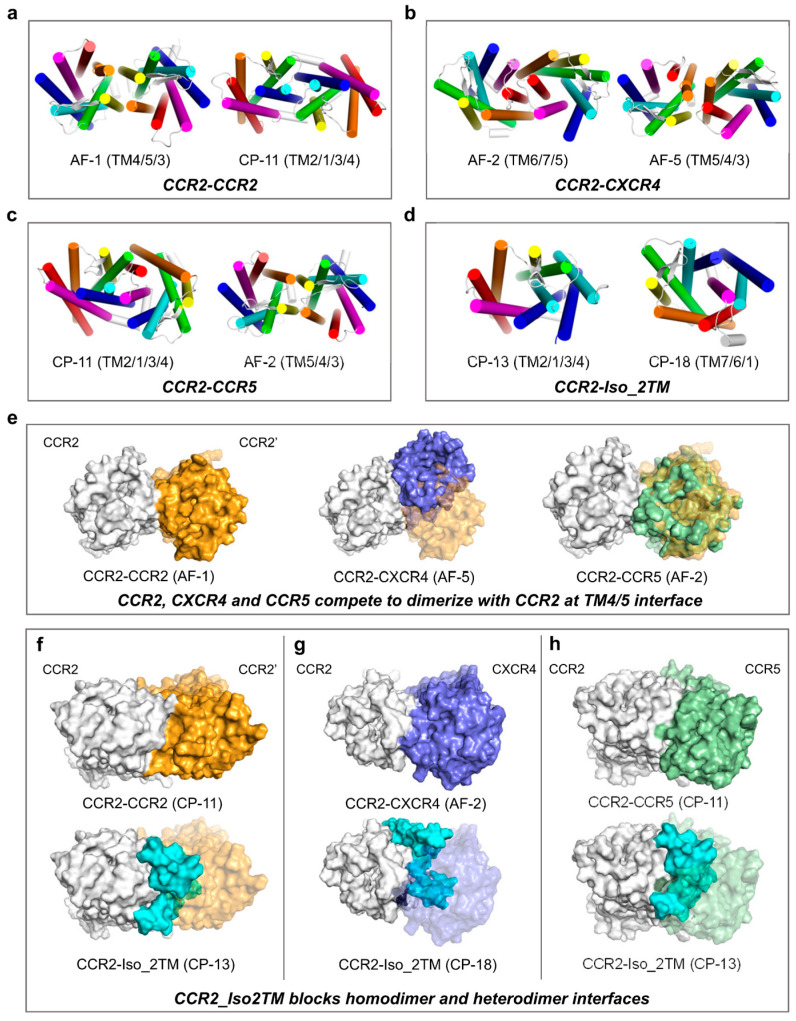
Interaction patterns of CCR2 homodimers, heterodimers, and complexes with its truncated isoforms: (**a**–**d**) Top view of the conformations of the most stable complex models of CCR2-CCR2 (**a**), CCR2-CXCR4 (**b**), CCR2-CCR5 (**c**), and CCR2-Iso_2TM (**d**), identified by binding free energy calculations. The display style is the same as that in Figure 2a. (**e**) CCR2, CXCR4, and CCR5 compete for dimerization with CCR2 at TM4/TM5 interface. The display style is the same as that in Figure 2f. (**f**–**h**) CCR2_Iso2TM blocks CCR2-CCR2 homodimer (**f**), CCR2-CXCR4 (**g**), and CCR2-CCR5 (**h**) heterodimer interfaces. The display style is the same as that in Figure 2f. All structural snapshots were taken from the medoid frame in the largest conformational cluster using PyMOL.

CCR2 can form homodimers [20,53,54] and heterodimers with CXCR4 [55,56] or CCR5 [57,58]. For each of these three dimers, we identified two models with distinct interfaces but with comparable binding strengths (Figure 5a–c). Notably, the binding strength of the CCR2 homodimer (BFE = ~−40 kcal/mol) is significantly lower than the moderately stable CCR2-CXCR4 heterodimer (~−100 kcal/mol) and highly stable CCR2-CCR5 heterodimer (<−120 kcal/mol), i.e., CCR2-CCR2 < CCR2-CXCR4 < CCR2-CCR5. Several previous studies indicated that CCR2 homodimerization requires induction by the ligand CCL2 [20,53,54]. In a study [14] based on the co-expression of CCR2, CXCR4, and CCR5 in HEK293 cells, CCR2-specific monoclonal antibody CCR2-01 induced CCR2 homodimerization, as it increased the homodimer amount from an undetectable level to a significantly detectable level, based on disuccinimidyl suberate crosslinking and CCR2 immunoprecipitation; by contrast, CCR2-01 further stabilized CCR2-CXCR4 and CCR2-CCR5 heterodimers, as it increased the heterodimer amount from a detectable level to a higher level, revealed via fluorescence resonance energy transfer (FRET). Under unstimulated conditions, CCR2-CCR5 heterodimers were notably more abundant than CCR2-CXCR4 heterodimers. These findings align well with our results. We found that TM4/TM5 (with limited TM3 contribution) could serve as the core interface for all three dimers (Figure 5e). Thus, at this interface, CCR2, CXCR4, and CCR5 could compete for dimerization with CCR2. Interestingly, at another interface, TM1/TM2 (with limited TM3/TM4 contribution), CCR2 and CCR5 could also compete for dimerization with CCR2 (Supplementary Figure S4). The two distinct interfaces of the CCR2 homodimer almost completely match the two interfaces (on CCR2) of the CCR2-CCR5 heterodimer, possibly due to their high sequence identity (79%; for reference, 33% between CCR2 and CXCR4) (Supplementary Figure S5).

A truncated isoform with two transmembrane helices (2TM) of CCR2 was identified, also composed of a complete N-terminus and truncated C-terminus (Supplementary Figure S1c). For the CCR2-Iso_2TM complex, we obtained two stable models with distinct interfaces (Figure 5d). Regarding the model with the TM1/TM2 interface, Iso-2TM (BFE = −149.04 ± 0.35 kcal/mol) could block CCR2 homodimerization (−39.61 ± 0.26 kcal/mol) and CCR2-CCR5 heterodimerization (−143.84 ± 0.24 kcal/mol) through a highly overlapping binding area and remarkably strong binding strength (Figure 5f,h). For the other model with the TM6/TM7 interface, Iso-2TM (BFE = −103.16 ± 0.22 kcal/mol) could block CCR2-CXCR4 heterodimerization (−100.08 ± 0.29 kcal/mol), albeit in a less strong manner (Figure 5h). Interestingly, similar to the CXCR2 truncated isoforms, either side of this CCR2 isoform could comprise the interface (Supplementary Figure S3c).

### 2.5. CCR7: Strong Blocking Capability of the Truncated Isoform

**Figure 6:**
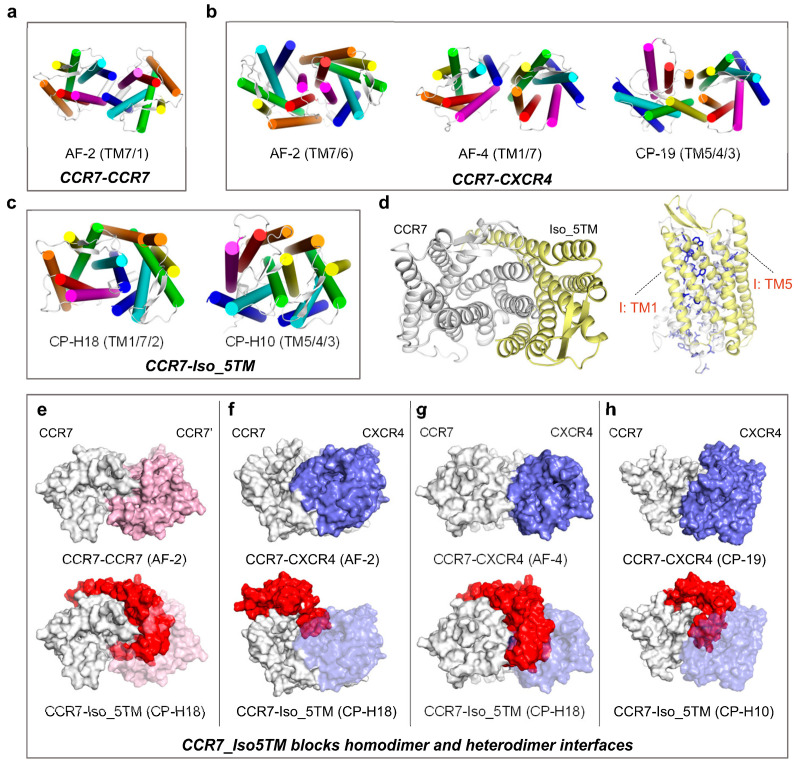
Interaction patterns of CCR7 homodimers, heterodimers, and complexes with its truncated isoforms: (**a**–**c**) Top view of the conformations of the most stable complex models of CCR7-CCR7 (**a**), CCR7-CXCR4 (**b**), and CCR7-Iso_5TM (**c**), identified by binding free energy calculations. The display style is the same as that in Figure 2a. (**d**) (Left) top view of the highly stable CCR7-Iso_5TM CP-H18 model. (Right) side view of the interfaces. The residues that positively contribute to interface formation are shown. The display style is the same as that in Figure 3. (**e**–**h**) CCR7_Iso5TM blocks CCR7-CCR7 homodimer (**e**) and CCR7-CXCR4 (**f**–**h**) heterodimer interfaces. The display style is the same as that in Figure 2f. All structural snapshots were taken from the medoid frame in the largest conformational cluster using PyMOL.

CCR7 can form homodimers [12,13,59] and heterodimers with CXCR4 [13,16,59]. Our results show that a model of the CCR7 homodimer exhibits significantly higher binding strength than other models within the same complex group (Supplementary Table S5). This model features the TM1/TM7 interface (with TM7 contributing predominantly), including the C-terminus Helix 8 (Figure 6a and Supplementary Figure S6). A study [12] combining crosslinking, homologous modeling, and mutant screening highlighted the critical role of TM7 and Helix 8 in CCR7 homodimer/oligomer formation, highly consistent with our results. For the CCR7-CXCR4 heterodimer, three models with distinct interfaces but with comparable binding strengths are obtained (Figure 6b). Two of these models adopt the TM6/TM7 and TM1/TM7 interfaces, respectively, both of which have overlapped portions with the CCR7 homodimer interface, suggesting competition between CCR7 and CXCR4 for dimerization with CCR7 at the TM7-centered interface.

CCR7 has a truncated isoform with five transmembrane helices (5TM), not only truncated at the C-terminus but also nearly completely truncated at the N-terminal loop (Supplementary Figure S1d). We obtained two CCR7-Iso_5TM models with markedly high binding strengths and distinct interfaces (Figure 6c). The model with the lowest BFE, CP-H18, exhibits the highest binding strength (−152.19 ± 0.29 kcal/mol) among all chemokine receptor–isoform complex models. The extensive binding area and massive residues actively contributing to interface formation reflect its tight binding (Figure 6d). This model adopts the TM1/TM7/TM2 interface, favorably overlapping with the interfaces in the CCR7 homodimer model (BFE = −118.45 ± 0.21 kcal/mol) and the abovementioned two CCR7-CXCR4 heterodimer models (−127.24 ± 0.26 kcal/mol and −119.23 ± 0.36 kcal/mol). Therefore, this indicates that Iso_5TM can block dimerization (Figure 6e–g). Furthermore, the other CCR7-Iso_5TM model (BFE = −140.47 ± 0.22 kcal/mol) shows that Iso_5TM can also block CCR7-CXCR4 heterodimerization (−117.10 ± 0.13 kcal/mol) at the TM3/TM4/TM5 interface (Figure 6h).

### 2.6. Possible Higher-Order Oligomerization Patterns of Chemokine Receptors

**Figure 7:**
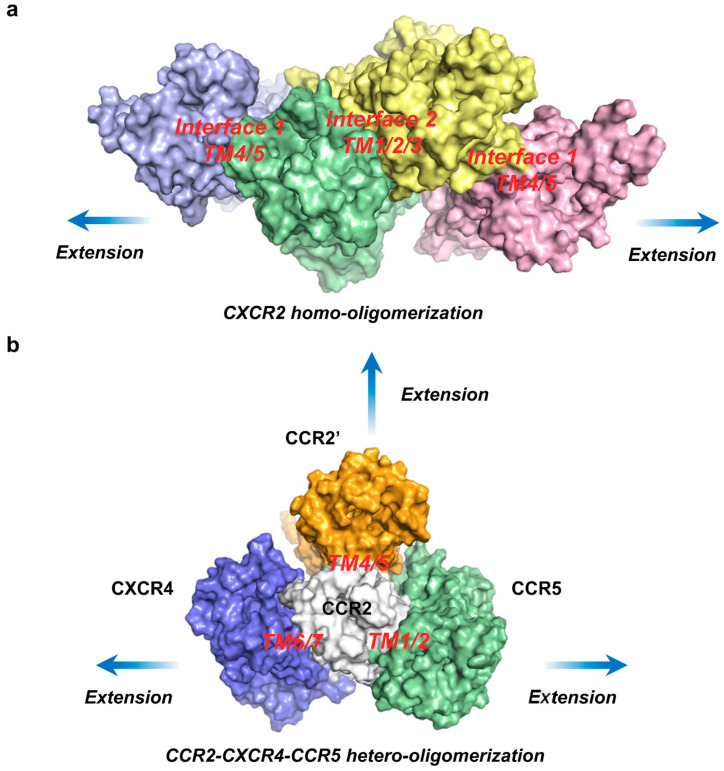
Putative oligomerization models, inferred from our resultant stable complex models” (**a**) Putative CXCR2 homo-oligomerization, shown as top view. Interface 1 (TM4/TM5) and Interface 2 (TM1/TM2/TM3) take turns, causing no clashes and being ready for continuous extension. (**b**) Putative CCR2-CXCR4-CCR5 hetero-oligomerization, shown in the top view. CCR2 first homodimerizes at the TM4/TM5 interface, binds CXCR4 at the TM6/TM7 interface, and binds CCR5 at the TM1/TM2 interface, causing no clashes and being ready for further extension. All structural snapshots were taken from the medoid frame in the largest conformational cluster using PyMOL.

Similar to many other GPCRs, the higher-order oligomerization of chemokine receptors has been reported [60,61], although its investigation is less intensive than dimerization, assumably due to technical challenges. Based on our results, we propose potential patterns for higher-order oligomerization using examples of the homo-oligomerization of CXCR2 (Figure 7a) and the hetero-oligomerization of CCR2 (Figure 7b). For CXCR2 homo-oligomerization, we present a model featuring two alternating interfaces, closely resembling the previously reported linear tetrameric structures of the µ-opioid receptor [62] and β1-adrenergic receptor [63], with no clashes and poised for continuous extension. It is reported that CCR2, CXCR4, and CCR5 can form ternary hetero-oligomers [60]. For CCR2’s hetero-oligomerization, we propose a model featuring a central CCR2 interacting with another CCR2, as well as CXCR4 and CCR5 simultaneously, with no clashes and ready for further extension along three directions.

## 3. Discussion

### 3.1. Dynamic and Diverse Dimerization Patterns and Higher-Order Oligomerization of Chemokine Receptors

The dynamics and diversity of GPCR dimerization patterns have been well reviewed [7,18,24], and for chemokine receptors, particularly CXCR4 and CCR5, this topic has been intensively studied [3,10]. Among chemokine receptors, little sequence conservation has been found in the residues comprising the dimerization site [64], even though oligomerization has been validated for many chemokine receptors. Our results reveal that the majority of the studied dimers exhibit two or three distinct dimerization patterns, and dimerization interface composition is considerably diverse across different dimer groups, suggesting the dynamic and diverse nature of the dimerization of these chemokine receptors. The different dimerization patterns of a GPCR may correspond to different functional states [18,65,66,67,68]. Based on this view, we infer that the functional implications of the dynamics of GPCR dimerization can be understood in terms of at least two aspects. Firstly, it allows for different signaling patterns in response to dynamic environmental changes and signals. Secondly, for higher-order oligomerization, additional functions need to be performed. Higher-order oligomerization requires the presence of diverse dimerization interfaces to extend the oligomeric structure, which is supported by our results.

### 3.2. Competitive Dimerization of Chemokine Receptors

Homodimerization and heterodimerization can occur simultaneously, forming higher-order oligomers. On the other hand, they can also conflict, competing at the overlapping interface. The previous study shows a competitive relationship between CXCR2 homodimerization and heterodimerization with CXCR1 and different levels of functional performance as the dimerization mode changes [19]. The ligand CXCL8 abolishes FRET for CXCR1/CXCR2 heterodimers and stabilizes FRET values for both homodimeric forms, suggesting dynamic dimerization, which is regulated by the ligand [19]. In addition to CXCR2, CXCR7 [52], CCR2 [54], and CCR7 [59] all showed distinct levels of functional performance when homodimerized and heterodimerized. Our findings demonstrate competitive dimerization across all e four chemokine receptors studied, suggesting that these chemokine receptors might switch between different dimerization forms to regulate their functions.

### 3.3. Predicted Inhibitory Effect of Truncated Isoforms on Dimerization

Some GPCR truncation isoforms have been demonstrated to form heterodimers with their full-length counterparts, resulting in retention in the endoplasmic reticulum or other organelles and the disability of functioning on the cell surface [38,39,40,41,42], and/or interfering with ligand binding [39,40,43]. A common feature of these truncation isoforms is C-terminal truncation, which is also featured by all the isoforms studied in this study. Previous research indicated that GPCR oligomerization occurs in the endoplasmic reticulum [49,69,70] and tends to be essential for the cell surface delivery of class A and class C GPCRs [9]. In addition, some similar truncated variants have shown the capability of competing for dimer formation with full-length counterparts [13,48]. It is reasonable to assume that, to achieve their inhibitory effects, these truncation isoforms would bind the full-length counterparts with similar or stronger binding affinity and overlapping interfaces, compared with the functional dimers. Our results demonstrate that the majority of the studied truncated isoforms meet these criteria; therefore, they can block more than one type of dimerization, strongly indicating their potential inhibitory effect on normal dimerization. Furthermore, these truncation isoforms exhibit consistently robust binding to their full-length counterparts and distinct interacting patterns compared with homodimers, which we speculate might be an outcome of evolution, as a mechanism of dynamic and fine regulation of the receptor function.

### 3.4. Combination of AlphaFold2 and Docking Enables More Comprehensive Interaction Pattern Exploration

Given the increasing interest in GPCR dimerization, computational studies about predicting GPCR dimerization interfaces have been emerging in the last two decades. These methods used for interface prediction are mainly based on molecular docking, molecular modeling, and coarse-grained molecular dynamics (CG-MD) simulations [24,71,72], all of which are based on physics. However, due to current computational capacity limitations and systematic error, the accurate and comprehensive exploration of conformational space remains challenging. The emergence of the neural network-based AlphaFold2 [27] offers an alternative approach for complex structure prediction, with its modified version AlphaFold-Multimer exhibiting exceptional prediction quality [29,30,31,73,74]. Recent work has shown that combining AlphaFold2 and the docking tool ClusPro through a nested-like structure can significantly enhance the success rate of complex structure prediction [75]. Our study utilized the outputs from both approaches in parallel as initial models for further binding strength assessment. Our results demonstrated that the two methods can yield models with consistent or distinct interaction patterns, and the most stable models can originate from either one or both approaches, albeit with a higher proportion from ClusPro. This highlights the capability of integrating these two fundamentally distinct methodologies to achieve a more comprehensive performance on complex structure prediction.

### 3.5. Suggested Systematic Experimental Studies

Our computation-based results present the most likely interfaces of homo- and heterodimers of four chemokine receptors and predict the presence and patterns of their interactions with their respective truncation isoforms. We would like to provide a few testable experimental suggestions: (i) constructing mutants based on our proposed interface information and assessing their dimerization propensity, along with chemical crosslinking [12,68], to explore dimer interface composition; (ii) investigating the co-expression of chemokine receptors and their truncation isoforms and validating their interactions through bioluminescence resonance energy transfer (BRET), fluorescence resonance energy transfer (FRET), or co-immunoprecipitation (Co-IP).

## 4. Conclusions

To the best of our knowledge, our study presents the first systematic study into the interaction patterns between GPCRs and their truncated isoforms at the structural bioinformatic level. We predicted the presence of interactions between four chemokine receptors and their truncated isoforms. Additionally, we predicted the homo- and heterodimerization interfaces of these chemokine receptors, supporting the dynamics and diversity of dimerization patterns. These findings could provide insights and guidance for further research on the oligomerization of chemokine receptors and the roles of truncated isoforms. If future experiments can verify our bioinformatic findings, these truncated chemokine receptors can be developed as therapeutics. For instance, truncated chemokine receptors can be specifically delivered to tumor cells and tissues, reducing metastasis and migration, since many metastasis tumors highly express several chemokine receptors [76].

## 5. Materials and Methods

### 5.1. Complex Structure Model Generation Using AlphaFold-Multimer and Structure Processing

The structure prediction of all complexes was performed using AlphaFold-Multimer [28] via the ColabFold [77] pipeline applying mostly default parameters. Briefly, the “alphafold2_multimer_v3” model type was applied; all five structures were relaxed; and “templates” mode was applied to maximize the similarity between predicted structures and experimental structures of protomers. The input sequences were from the UniProt database (https://www.uniprot.org/, accessed on 6 October 2023), with entries summarized in Supplementary Table S1. In Supplementary Figure S3, the alignment figures were created using the UniProt “align” tool; the topological figures were created using the Protter server [78]. After the structure models were generated, we selected some of them and processed these models for MD simulations. The AlphaFold2 prediction confidence profiles of the models in Table 1 are summarized in Supplementary Figure S7. For each complex, we selected the models with unique interfaces; if all models showed similar interfaces, we selected two models with relatively distinct interfaces. For each selected model, we aligned the protomer structure with its experimental structure and replaced the predicted structure with the experimental structure. Regarding truncated isoforms, we did not make modifications as there were no available experimental structures. For experimental structures, we used 8IC0 (PDB entry) for CXCR1, 6LFM for CXCR2, 3ODU for CXCR4, 7SK3 for CXCR7, 7XA3 for CCR2, 7O7F for CCR5 and 6QZH for CCR7 and exported the chemokine receptor structures for further use. For 3ODU (CXCR4) and 6QZH (CCR7), the mutations constructed for crystallization and the missing loops connecting helices were repaired by inputting the original sequence and homogenous modeling using PyMod 3 [79]. For 3ODU (CXCR4), the C-terminal loop (residue 277–302) was removed as it significantly blocked binding with the other protomer. Steric clashes, if present, were solved by rotating the clashing side chains or translating the whole protomer structure, using PyMOL (version 2.5, Schrödinger, NY, USA), until no steric clashes existed. The resultant structures were used as the inputs of MD simulations.

### 5.2. Protein–Protein Docking Using ClusPro and Structure Processing

The protein–protein docking of all complexes was performed using the ClusPro server [46] applying all default parameters. Experimental structures of chemokine receptors and AlphaFold2 models of truncated isoforms (the PDB entries and structure processing same as abovementioned) were used as inputs of docking. For the resultant models, we selected “topology-compliant” models from 30 models obtained with the default “Balanced mode” and 30 models obtained with the “Hydrophobic-favored mode”. “Topology-compliant” models are defined when the following rules are met: (i) The orientation of both protomers is consistent with a typical GPCR orientation; (ii) the putative membrane layers inferred from transmembrane regions of both protomers are basically spatially consistent; (iii) if it is a homodimer model, the interface composition of both protomers is identical. The selected models were used as the inputs of MD simulations.

### 5.3. Molecular Dynamics Simulations

The membrane–protein systems were built using the membrane builder [80] of the CHARMM-GUI web server [81]. The protein portion was centered in a rectangular box, with an X/Y length of 12 nm for dimers and 11 nm for complexes containing truncated isoforms. The membrane consists of 70% 1-palmitoyl-2-oleoyl-glycero-3-phosphocholine (POPC) and 30% cholesterol. The system was solvated in TIP3P water with 150 mM KCl.

All MD simulations were performed using GROMACS 2022.3 [82]. The all-atom CHARMM36m force field was used. The system energy was minimized using the steepest descent method, and the maximum forces converged below 1000 kJ/mol/nm. Electrostatics were treated with particle–mesh Ewald method, and the cutoff for both Coulomb and van der Waals interactions was 1.2 nm. Then, 125 ps equilibration simulations were performed using the standard six-step CHARMM-GUI protocol [83]. After NVT and NPT equilibration, a 20 ns further equilibration simulation was run to achieve the stabilization of the complexes, which was assessed using the RMSD and trajectory of the complex. This was followed by a 50 ns production MD simulation, which was subsequently subjected to binding free energy calculations. These 70 ns simulations were performed under the following setting: 2 fs time step was used with the SHAKE algorithm. The van der Waals interactions were smoothly switched off at 10–12 Å using a force-switching function. Long-range electrostatic interactions were calculated using the particle–mesh Ewald method. The Parrinello–Rahman barostat was used with semi-isotropic coupling and the Nose–Hoover thermostat was used. Temperature was held at 303.15 K, and pressure was held at 1 bar, respectively.

For conformational analysis, simulation frames were extracted every 250 ps and clustered (GROMACS built-in command “gmx cluster”) using the linkage method with a cutoff that produced a minimal number of clusters. The medoid structure of the largest cluster was exported and used for further analysis. The structure figures were created using PyMOL or ChimeraX [84].

### 5.4. Binding Free Energy Calculation

The binding free energy of the complexes was calculated using the Molecular Mechanics Poisson–Boltzmann Surface Area (MMPBSA) algorithm and performed using the gmx_MMPBSA tool [47]. For each model, all 5001 frames of the 50 ns simulation were subjected to calculations. The calculations were performed under a setting consistent with the recommended setting for the protein–membrane systems prepared using CHARMM force fields (https://valdes-tresanco-ms.github.io/gmx_MMPBSA/v1.5.6/examples/Protein_membrane_CHARMMff/, accessed on 6 October 2023). The setting was as follows: Poisson–Boltzmann (PB) calculations were carried out; the temperature was 303.15 K; the PB radii were “charmm_radii” (PBRadii = 7); a uniform membrane dielectric constant in a slab-like implicit membrane was used (memopt = 1); the membrane dielectric constant was 7.0; radii from the prmtop file for both the PB calculation and for the nonpolar calculation were used (radiopt = 0); ionic strength was 0.15 M; the ratio between the longest dimension of the rectangular finite-difference grid and that of the solute was 1.25 (fillratio = 1.25); the total nonpolar solvation free energy was modeled as a single term linearly proportional to the solvent accessible surface area (inp = 1); a geometric multigrid was used for iterative solvers (solvopt = 2); a classical geometric method was used for setting up a dielectric model for all numerical PB procedures (ipb = 1); periodic boundary condition was used (bcopt = 10); no focusing was used (nfocus = 1); computing total electrostatic energy and forces with the particle–particle particle–mesh (P3M) procedure outlined in Lu and Luo [85] was applied (eneopt = 1); atom-based cutoff distance for removing short-range finite-difference interactions and adding pairwise charge-based interactions was 7.0 nm (cutfd = 7.0); the cutoff distance used for van der Waals interactions was 99.0 nm (cutnb = 99.0); SASA was used to estimate cavity free energy (use_sav = 0); number of dots used to store arc dots per atom was 15,000 (maxarcdot = 15,000); verbose mode was on (npbverb = 1); all residues within 5 Å between receptor and ligand were printed. For the analysis of residues contributing to binding, the gmx_mmpbsa_ana tool was used.

## Figures and Tables

**Table 1:** Summary of dimerization interfaces and binding free energy calculations of the most stable complex models.

Complex (Subject–Partner)	Model	Interface Composition		Binding Free Energy (kcal/mol)
		Subject (Left)	Partner (Right)	
**CXCR2-CXCR2**	AF-3	TM4, TM5	Identical	−70.52 ± 0.14
	CP-12	TM1, TM2, TM3 (E)	Identical	−69.82 ± 0.33
**CXCR2-CXCR1**	CP-H18	TM7, TM6, TM1	TM4, TM5	−118.83 ± 0.19
	CP-14	TM1, TM2, TM3 (E)	TM7, TM1, TM6	−110.32 ± 0.23
**CXCR2-Iso_2TM**	CP-17	TM6, TM7, TM1	TM2, TM1	−133.80 ± 0.18
	CP-16	TM4, TM5	TM1, TM2	−104.69 ± 0.29
**CXCR2-Iso_3TM**	CP-10	TM7, TM1, TM6	TM2, TM1, TM3 (E)	−123.76 ± 0.22
**CXCR2-Iso_4TM**	AF-5	TM6, TM7 (E)	TM1, TM2 (E), TM3 (E)	−105.62 ± 0.19
	CP-28	TM5, TM4, TM6	TM1, TM2, TM3	−96.96 ± 0.20
**CXCR7-CXCR7**	CP-H8	TM6, TM7, TM1 (M)	Identical	−160.12 ± 0.41
**CXCR7-CXCR4**	CP-19	TM7, TM6, TM1	TM5, TM4	−150.17 ± 0.21
**CXCR7-Iso_4TM**	CP-H14	TM2, TM1, TM3 (E), TM4 (M)	TM1, TM2, TM3	−104.81 ± 0.16
**CCR2-CCR2**	AF-1	TM4, TM5, TM3 (I)	Identical	−44.84 ± 0.16
	CP-11	TM2, TM1, TM3 (E), TM4(I)	Identical	−39.61 ± 0.26
**CCR2-CXCR4**	AF-2	TM6, TM7, TM5	TM6, TM5	−100.08 ± 0.29
	AF-5	TM5, TM4, TM3 (I)	Identical	−98.45 ± 0.14
**CCR2-CCR5**	CP-11	TM2, TM1, TM3 (E), TM4 (I)	TM7, TM1, TM6	−143.84 ± 0.24
	AF-2	TM5, TM4, TM3 (I)	Identical	−121.76 ± 0.14
**CCR2-Iso_2TM**	CP-13	TM2, TM1, TM3 (E), TM4 (I)	TM1, TM2	−149.04 ± 0.35
	CP-18	TM7, TM6, TM1	TM1, TM2	−103.16 ± 0.22
**CCR7-CCR7**	AF-2	TM7, TM1	Identical	−118.45 ± 0.21
**CCR7-CXCR4**	AF-2	TM7, TM6	TM6, TM7	−127.24 ± 0.26
	AF-4	TM1, TM7 (I)	TM4, TM5	−119.23 ± 0.36
	CP-19	TM5, TM4, TM3 (I)	Identical	−117.10 ± 0.13
**CCR7-Iso_5TM**	CP-H18	TM1, TM7, TM2	TM2, TM1, TM3, TM5	−152.19 ± 0.29
	CP-H10	TM5, TM4, TM3(E)	TM5, TM4, TM1, TM2	−140.47 ± 0.22

Note: The content of “interface composition” is ordered by the contribution to the interface formation, and only TM helices are shown. For example, “TM7, TM1, TM6” indicates TM7 > TM1 > TM6 in terms of contact contribution; E, the extracellular side-facing part of the helix; M, the middle part of the helix; I, the intracellular side-facing part of the helix. No parenthesis indicates that the majority of this helix is involved in the interface composition. “AF-1” denotes the Rank 1 model generated using AlphaFold-Multimer; “CP-12” denotes the No.12 model generated using ClusPro with the default Balanced mode, and “CP-H12” denotes the No.12 model generated using ClusPro with the Hydrophobic-favored mode. Isoforms are displayed in abbreviated forms without prefixes. For example, “CXCR2-Iso_2TM” denotes the complex formed by CXCR2 and its truncated isoform with two transmembrane helices. Binding free energy values are shown as mean ± standard error of the mean (SEM).

## Data Availability

All data and code have been deposited in the Zenodo database (DOI: 10.5281/zenodo.8393388). Further information and requests for data should be directed to and will be fulfilled by S.Z. (shuguang@mit.edu).

## Supplementary Materials

The supporting information can be downloaded at: https://www.mdpi.com/article/10.3390/ijms242216266/s1.
