# Supplementary material for: Dynamic Dimerization of Chemokine Receptors and Potential Inhibitory Role of Their Truncated Isoforms Revealed through Combinatorial Prediction
Source: Int J Mol Sci. 2023 Nov 13;24(22):16266. doi: 10.3390/ijms242216266 (PMC10671024; doi:10.3390/ijms242216266)
Supplement: Supplementary file 1 [file ijms-24-16266-s001.zip › ijms-2676413-supplementary.pdf]

Supplementary Material

Dynamic dimerization of chemokine receptors and potential inhibitory role of their truncated isoforms revealed by combinatorial prediction

Mengke Li, Rui Qing, Fei Tao, Ping Xu, Shuguang Zhang

*Corresponding author: Shuguang Zhang
Email: shuguang@mit.edu

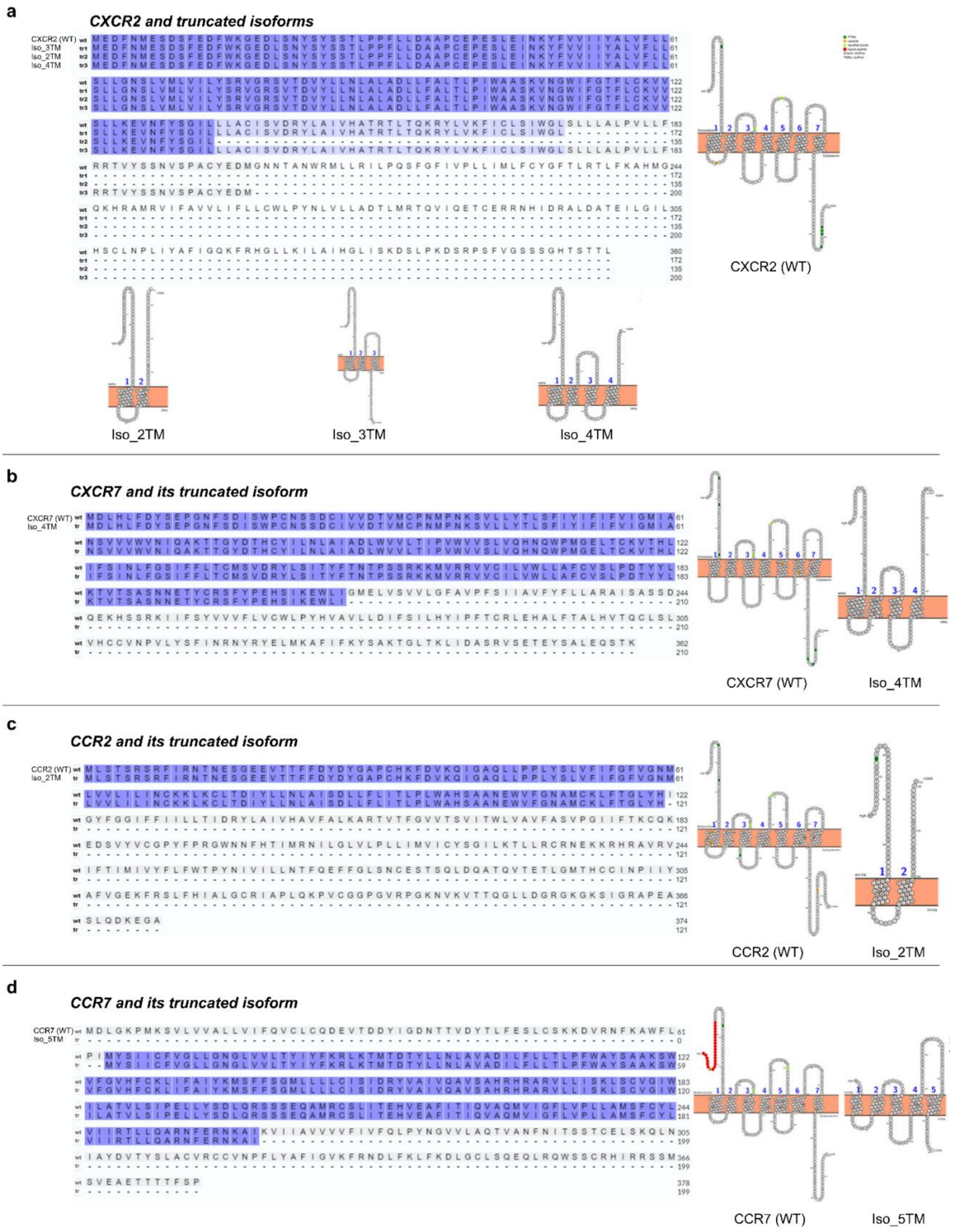

Supplementary Figure S1. Sequence alignment between chemokine receptors and their truncated isoforms and their predicted topologic structures. (a – d) CXCR2, CXCR7, CCR2 and CCR7, respectively. The alignment figures (left) were made using Uniprot “align” tool. The topological figures (right) were made using the Protter server.

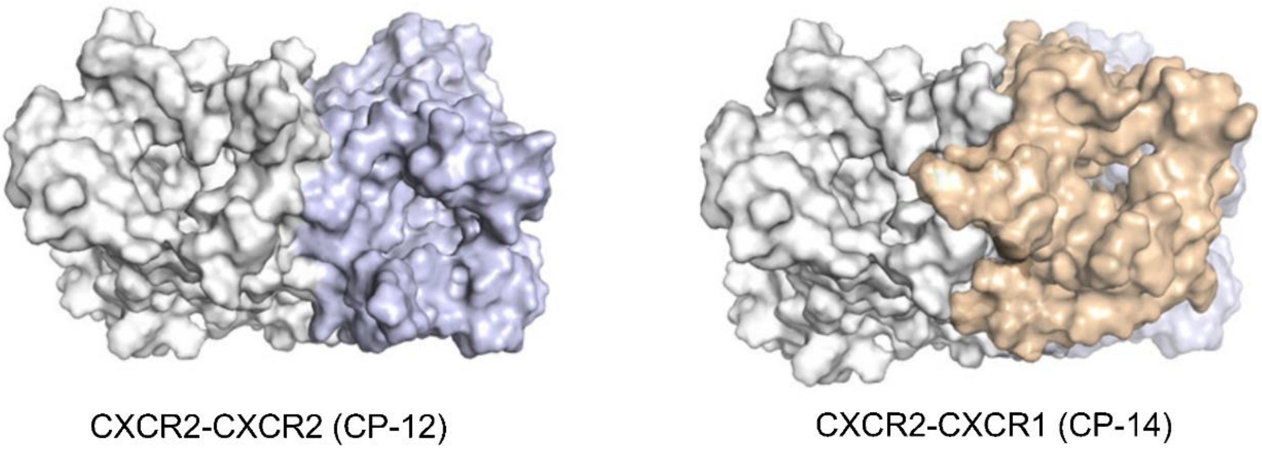

Supplementary Figure S2. CXCR2 and CXCR1 compete to dimerize with CXCR2 at TM1/TM2/TM3 interface. The display style is the same as Figure 2f.

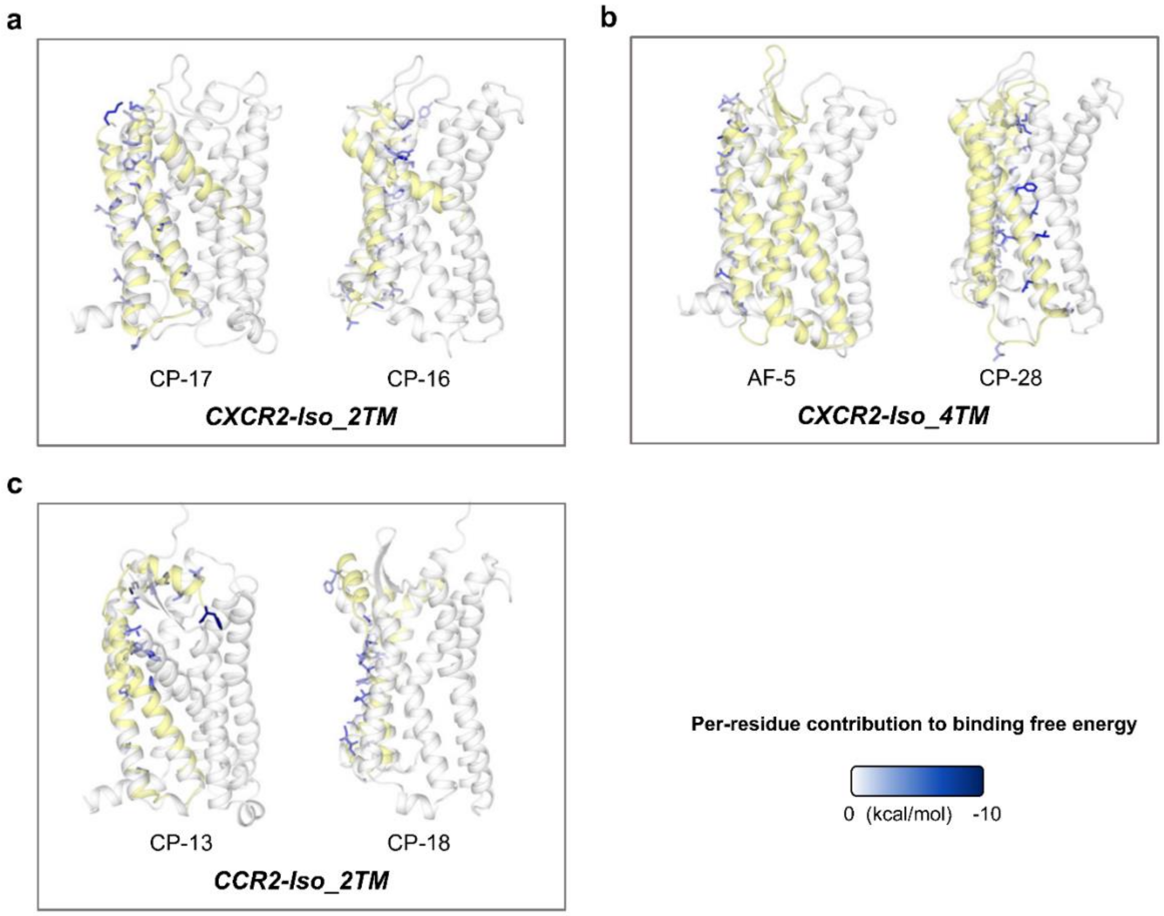

Supplementary Figure S3. Either side of truncated isoforms could comprise the interface. For each shown model, the truncated isoform structure (yellow) with the residues (gradient blue) that contribute to interface formation were aligned to the experimental structure of their full-length counterparts (white). All structure snapshots were made using PyMOL.

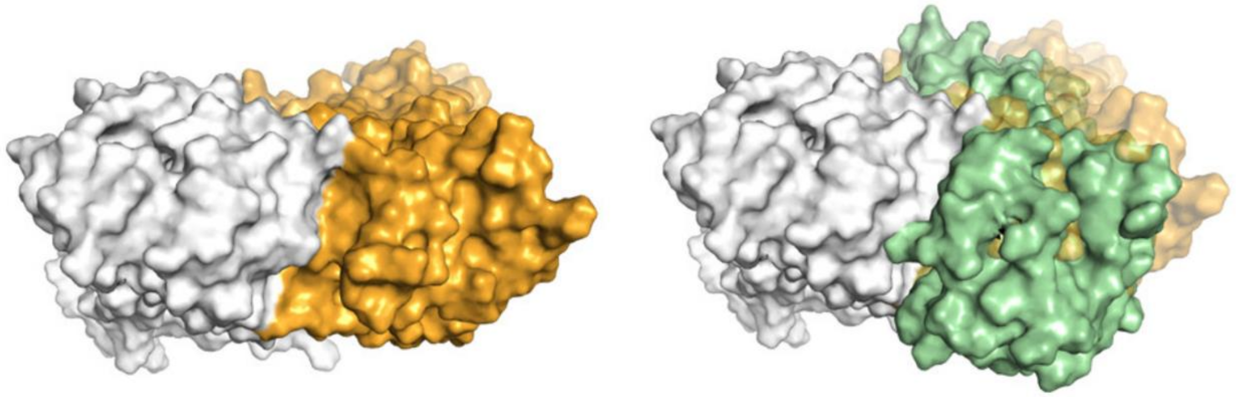

CCR2-CCR2 (CP-11) CCR2-CCR5 (CP-11)

Supplementary Figure S4. CCR2 and CCR5 compete to dimerize with CCR2 at TM1/TM2 interface. The display style is the same as Figure 2f.

a

Score	Expect	Method	Identities	Positives	Gaps
452 bits(1164)	1e-164	Compositional matrix adjust.	231/294(79%)	256/294(87%)	4/294(1%)

```
CCR2  Query  25   DYDYGAPCHKFDVKQIGAQLLPPLYSLVFIFGFVGNMLVVLILINCKKLKCLTDIYLLNL  84
                  +Y    PC K +VKQI A+LLPPLYSLVFIFGFVGNMLV+LILINCK+LK +TDIYLLNL
CCR5  Sbjct  13   NYYTSEPCQKINVKQIAARLLPPLYSLVFIFGFVGNMLVILILINCKRLKSMTDIYLLNL  72

      Query  85   AISDLLFLITLPLWAHSAANEWVFGNAMCKLFTGLYHIGYFGGIFFIILLTIDRYLAIVH  144
                  AISDL FL+T+P WAH AA +W FGN MC+L TGLY IG+F GIFFIILLTIDRYLA+VH
      Sbjct  73   AISDLFFLLTVPFWAHYAAAQWDFGNTMCQLLTGLYFIGFFSGIFFIILLTIDRYLAVVH  132

      Query  145  AVFALKARTVTFGVVTSVITWLVAVFASVPGIIFTKCQKEDSVYVCGPYFPRG----WNN  200
                  AVFALKARTVTFGVVTSVITW+VAVFAS+PGIIFT+ QKE   Y C  +FP      W N
      Sbjct  133  AVFALKARTVTFGVVTSVITWVVAVFASLPGIIFTRSQKEGLHYTCSSHFPYSQYQFWKN  192

      Query  201  FHTIMRNILGLVLPLLIMVICYSGILKTLLRCRNEKKRHRAVRVIFTIMIVYFLFWTPYN  260
                  F T+   ILGLVLPLL+MVICYSGILKTLLRCRNEKKRHRAVR+IFTIMIVYFLFW PYN
      Sbjct  193  FQTLKIVILGLVLPLLVMVICYSGILKTLLRCRNEKKRHRAVRLIFTIMIVYFLFWAPYN  252

      Query  261  IVILLNTFQEFFGLSNCESTSQLDQATQVTETLGMTHCCINPIIYAFVGEKFRS  314
                  IV+LLNTFQEFFGL+NC S+++LDQA QVTETLGMTHCCINPIIYAFVGEKFR+
      Sbjct  253  IVLLLNTFQEFFGLNNCSSSNRLDQAMQVTETLGMTHCCINPIIYAFVGEKFRN  306
```

b

Score	Expect	Method	Identities	Positives	Gaps
195 bits(496)	7e-64	Compositional matrix adjust.	108/330(33%)	180/330(54%)	9/330(2%)

```
CCR2   Query  13   TNESGEEVTTFFDYD-YGAPCHKFDVKQIGAQLLPPLYSLVFIFGFVGNMLVVLILINCK  71
                   T+++  E     DYD    PC + +        LP +YS++F+ G VGN LV+L++   K
CXCR4  Sbjct  8    TSDNYTEEMGSGDYDSMKEPCFREENANFNKIFLPTIYSIIFLTGIVGNGLVILVMGYQK  67

       Query  72   KLKCLTDIYLLNLAISDLLFLITLPLWAHSAANEWVFGNAMCKLFTGLYHIGYFGGIFFI  131
                   KL+ +TD Y L+L+++DLLF+ITLP WA  A   W FGN +CK    +Y +  +  +  +
       Sbjct  68   KLRSMTDKYRLHLSVADLLFVITLPFWAVDAVANWYFGNFLCKAVHVIYTVNLYSSVLIL  127

       Query  132  ILLTIDRYLAIVHAVFALKARTVTFGVVTSVITWLVAVFASVPGIIFTKCQKEDSVYVCG  191
                     +++DRYLAIVHA  + + R +    V  V  W+ A+  ++P  IF    + D  Y+C
       Sbjct  128  AFISLDRYLAIVHATNSQRPRKLLAEKVVYVGVWIPALLLTIPDFIFANVSEADDRYICD  187

       Query  192  PYFPRG-WNNFHTIMRNILGLVLPLLIMVICYSGILKTLLRCRNEKKRHRAVRVIFTIMI  250
                    ++P   W         ++GL+LP ++++ CY  I+  L   +  +KR +A++    +++
       Sbjct  188  RFYPNDLWVVVFQFQHIMVGLILPGIVILSCYCIIISKLSHSKGHQKR-KALKTTVILIL  246

       Query  251  VYFLFWTPYNIVILLNTFQEFFGLSN-CESTSQLDQATQVTETLGMTHCCINPIIYAFVG  309
                    +F  W PY I I +++F     +   CE  + + +   +TE L   HCC+NPI+YAF+G
       Sbjct  247  AFFACWLPYYIGISIDSFILLEIIKQGCEFENTVHKWISITEALAFFHCCLNPILYAFLG  306

       Query  310  EKFR-----SLFHIALGCRIAPLQKPVCGG  334
                    KF+     +L  ++ G  +  L K   GG
       Sbjct  307  AKFKTSAQHALTSVSRGSSLKILSKGKRGG  336
```

Supplementary Figure S5. Sequence alignment between CCR2 and CCR5 (a) or CXCR4 (b). The alignment was done using BLAST tool of NCBI.

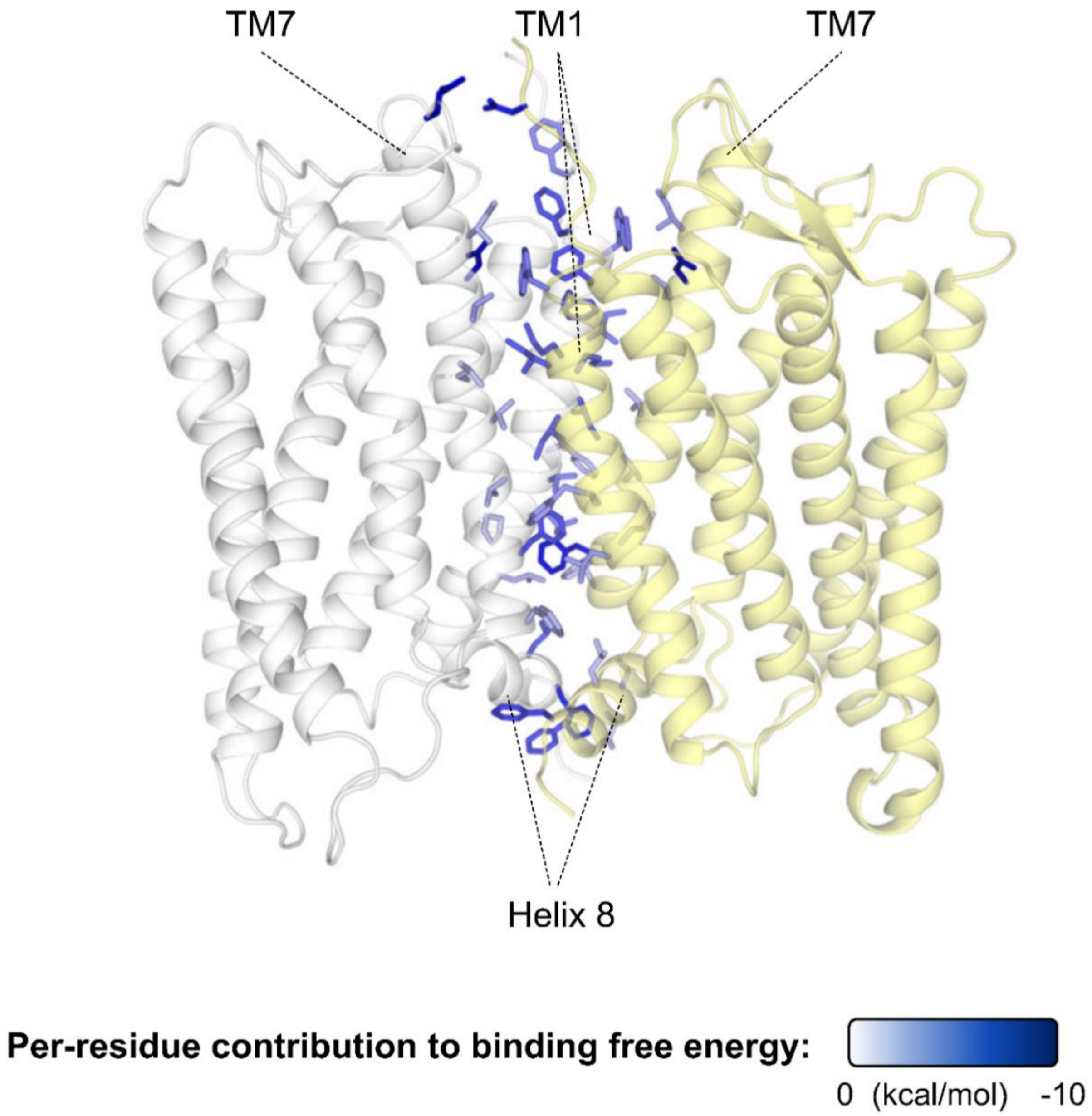

Supplementary Figure S6. Side view of the interface of CCR7-CCR7 (AF-2). The residues that contribute positively to interface formation are shown. The display style is the same as Figure 3.

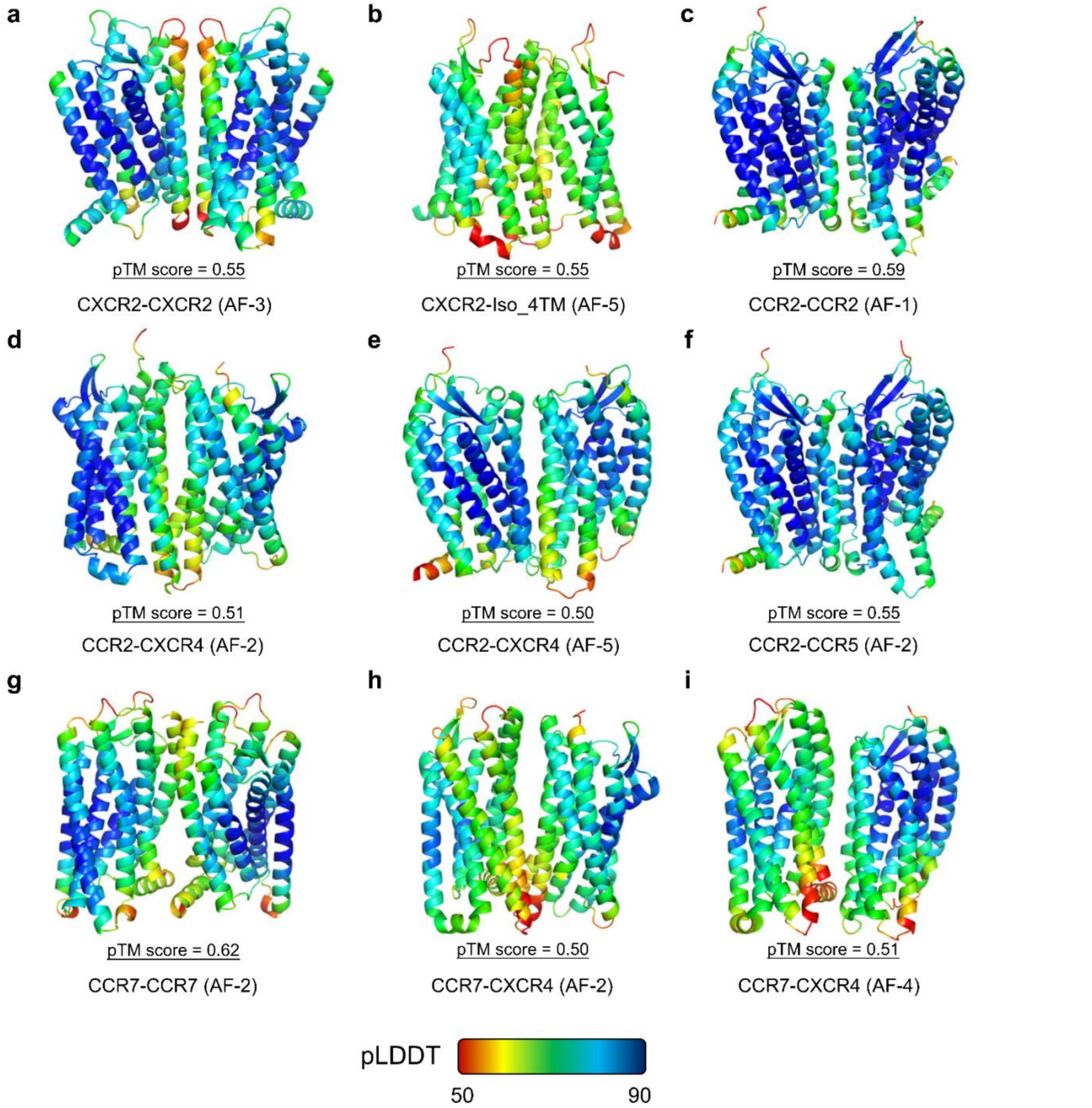

Supplementary Figure S7. Prediction confidence of AlphaFold2 models. (a – i) predicted Local Distance Difference Test (pLDDT) scores for assessing local structure confidence and predicted Template Modeling score (pTM score) for assessing protein–protein interaction. The pLDDT scale was shown at the bottom. To provide a relative reference for pTM scoring, we predicted the structure of the CXCR4 dimer, whose experimental structure is already available and in the training dataset of AlphaFold2. The average PTM score was 0.60.

Table S1. Human CC and CXC chemokine receptors and their truncated isoforms

Name	Available Structure?	Isoform type	Length (aa)	TM number	Uniprot	Existence level[a]	GPCRdb isoform
CCR1	Yes	Reference	355	7	P32246		
**CCR2**	**Yes**	Reference	374	7	P41597		
		**Truncated**	**121**	**2**	E9PH76	Protein	ENST00000447924
CCR3	Yes	Reference	355	7	P51677		
		Truncated	80	1	Q8TDP5	Transcript	Not found
CCR4	No	Reference	360	7	P51679		
CCR5	Yes	Reference	352	7	P51681		
CCR6	Yes	Reference	374	7	P51684		
**CCR7**	**Yes**	Reference	378	7	P32248		
		**Truncated**	**199**	**5**	J3KTN5	Protein	**Not found**
CCR8	No	Reference	355	7	P51685		
		Truncated	272	6	P51685-2	Protein	ENST00000545843
CCR9	Yes	Reference	369	7	P51686		
CCR10	No	Reference	362	7	P46092		
		Truncated	83	1	K7EPC9	Protein	ENST00000591568
		Truncated	140	2	K7ER70	Protein	ENST00000591765
CCR11	No	Reference	344	7	O00421		
		Truncated	217	5	C9JP23	Protein	ENST00000433848
CXCR1	Yes	Reference	350	7	P25024		
**CXCR2**	**Yes**	Reference	360	7	P25025		
		**Truncated**	**200**	**4**	C9JW47	Protein	ENST00000453237
		**Truncated**	**172**	**3**	C9J1J7	Protein	ENST00000428565
		**Truncated**	**135**	**2**	C9JG19	Protein	ENST00000454148
		Truncated[b]	138	2	C9J2F9	Protein	ENST00000415392
CXCR3	No	Reference	368	7	P49682		
		Truncated	267	4	P49682-3	Protein	Not found
CXCR4	Yes	Reference	352	7	P61073		
CXCR5	No	Reference	372	7	P32302		
CXCR6	No	Reference	342	7	O00574		
**CXCR7**	**Yes**	Reference	362	7	P25106		
		**Truncated**	**210**	**4**	A0A140T9K6	Protein	ENST00000447924

a: The type of evidence that supports the existence of the protein, according to Uniprot database.
b: The **bolded** text indicates the subjects selected in this study. The 138-aa CXCR2 truncated isoform is not selected because it is only three amino acids longer than the 135-aa one.

Table S2. Summary of dimerization interfaces and BFE calculations of the complex structure models, for CXCR2-related complexes

Complex	Model source	Model	Interface composition		Binding free energy (kcal/mol)
			CXCR2	Partner	
**CXCR2-CXCR2**	AF2	AF-1	TM1	Identical	-46.43 ± 0.13
		**AF-3**	**TM4, TM5**	**Identical**	**-70.52 ± 0.14**
		AF-4	TM6, TM5 (I), TM7 (E)	Identical	-39.43 ± 0.14
	Docking	**CP-12**	**TM1, TM2, TM3 (E)**	**Identical**	**-69.82 ± 0.33**
		CP-29	TM5, TM6	Identical	-59.34 ± 0.24
**CXCR2-CXCR1**	AF2	AF-1	TM4, TM5, TM3 (I)	Identical	-57.29 ± 0.20
		AF-2	TM4, TM5, TM3 (I)	Identical	-71.37 ± 0.26
	Docking	CP-6	TM5, TM4	TM7, TM6, TM1	-73.65 ± 0.30
		**CP-14**	**TM1, TM2, TM3 (E)**	**TM7, TM1, TM6**	**-110.32 ± 0.23**
		CP-19	TM1, TM2, TM3 (E)	TM2, TM1, TM3(E)	-105.89 ± 0.15
		**CP-H18**	**TM7, TM6, TM1**	**TM4, TM5**	**-118.83 ± 0.19**
**CXCR2-Iso_2TM**	AF2	AF-1	TM6, TM7 (E), TM5 (I)	TM1, TM2 (E)	-74.77 ± 0.13
		AF-2	TM1, TM2	TM1, TM2 (E)	-41.16 ± 0.20
		AF-3	TM6, TM7 (M)	TM1	-32.59 ± 0.10
		AF-4	TM5, TM6 (E), TM3 (I)	TM1	-74.55 ± 0.09
	Docking	CP-6	TM6, TM7	TM1, TM2	-119.52 ± 0.21
		**CP-16**	**TM4, TM5**	**TM1, TM2**	**-104.69 ± 0.29**
		**CP-17**	**TM6, TM7, TM1**	**TM2, TM1**	**-133.80 ± 0.18**
		CP-25	TM1, TM7	TM1, TM2	-70.71 ± 0.17
**CXCR2-Iso_3TM**	AF2	AF-1	TM6, TM7 (E)	TM1, TM2 (E)	-86.05 ± 0.17
		AF-2	TM6, TM7 (E)	TM1	-67.42 ± 0.26
		AF-3	TM1, TM2 (E)	TM1, TM2 (E)	-67.82 ± 0.09
		AF-4	TM6, TM7, TM1 (E)	TM1	-77.77 ± 0.17
	Docking	**CP-10**	**TM7, TM1, TM6**	**TM2, TM1, TM3 (E)**	**-123.76 ± 0.22**
		CP-11	TM5, TM4, TM6 (M)	TM1, TM3, TM2	-34.25 ± 0.32
		CP-12	TM2, TM1, TM3 (E)	TM2, TM1, TM3 (E)	-76.39 ± 0.21
**CXCR2-Iso_4TM**	AF2	AF-1	TM6, TM5 (I), TM7 (E)	TM1, TM2 (I)	-28.23 ± 0.28
		AF-3	TM6, TM7 (E), TM1 (E)	TM1	-40.66 ± 0.18
		AF-4	TM4, TM5 (E)	TM4, TM2, TM3 (E)	-49.47 ± 0.20
		**AF-5**	**TM6, TM7 (E)**	**TM1, TM2 (E)**	**-105.62 ± 0.19**
	Docking	CP-11	TM1, TM2, TM3 (E)	TM1, TM3 (I)	-79.97 ± 0.12
		**CP-28**	**TM5, TM4, TM6**	**TM1, TM2, TM3**	**-96.96 ± 0.20**

Table S3. Summary of dimerization interfaces and BFE calculations of the complex structure models, for CXCR7-related complexes

Complex	Model source	Model	Interface composition		Binding free energy (kcal/mol)
			CXCR7	Interacting partner	
**CXCR7-CXCR7**	AF2	AF-1	TM1, TM2 (E)	Identical	-94.18 ± 0.10
		AF-2	TM4, TM5, TM3 (I)	Identical	-87.11 ± 0.17
		AF-5	TM5, TM3 (I), TM6 (E)	Identical	-63.14 ± 0.18
	Docking	**CP-H8**	**TM6, TM7, TM1 (M)**	**Identical**	**-160.12 ± 0.41**
**CXCR7-CXCR4**	AF2	AF-1	TM1, TM2 (E)	Identical	-68.78 ± 0.14
		AF-2	TM1, TM2 (E)	TM6, TM7, TM1 (E)	-57.71 ± 0.18
		AF-3	TM1, TM2 (E)	TM5, TM6 (E)	-67.92 ± 0.14
		AF-5	TM4, TM5, TM3 (I)	TM1, TM6, TM7	-115.42 ± 0.26
	Docking	**CP-19**	**TM7, TM6, TM1**	**TM5, TM4**	**-150.17 ± 0.21**
		CP-21	TM5, TM4	TM4, TM5	-122.04 ± 0.16
**CXCR7-Iso_4TM**	AF2	AF-1	TM1, TM2 (E)	TM1, TM2 (E)	-85.74 ± 0.10
		AF-4	TM4, TM3 (E)	TM4, TM3 (E), TM2 (I)	-65.75 ± 0.27
	Docking	CP-28	TM1, TM2 (E), TM3 (E)	TM1, TM3 (M)	-99.87 ± 0.25
		**CP-H14**	**TM2, TM1, TM3 (E), TM4 (M)**	**TM1, TM2, TM3**	**-104.81 ± 0.16**
		CP-H16	TM6, TM5, TM7	TM1, TM2, TM3	-90.88 ± 0.17

Table S4. Summary of dimerization interfaces and BFE calculations of the complex structure models, for CCR2-related complexes

Complex	Model source	Model	Interface composition		Binding free energy (kcal/mol)
			CCR2	Interacting partner	
CCR2-CCR2	AF2	**AF-1**	**TM4, TM5, TM3 (I)**	**Identical**	**-44.84 ± 0.16**
		AF-3	TM4, TM3 (E)	Identical	13.02 ± 0.21
		AF-4	TM6, TM5	Identical	-20.36 ± 0.29
	Docking	**CP-11**	**TM2, TM1, TM3 (E), TM4(I)**	**Identical**	**-39.61 ± 0.26**
		CP-15	TM4, TM5, TM3 (I)	Identical	-40.21 ± 0.25
CCR2-CXCR4	AF2	AF-1	TM4, TM5, TM3 (I)	Identical	-92.72 ± 0.14
		**AF-2**	**TM6, TM7, TM5**	**TM6, TM5**	**-100.08 ± 0.29**
		AF-3	TM6, TM5, TM7(E)	TM5, TM6	14.16 ± 0.21
		AF-4	TM4, TM5 (E)	TM5, TM6	-35.76 ± 0.14
		**AF-5**	**TM5, TM4, TM3 (I)**	**Identical**	**-98.45 ± 0.14**
	Docking	CP-22	TM6, TM5	TM5, TM4 (E)	-50.92 ± 0.16
		CP-H9	TM5, TM6	Identical	-71.30 ± 0.30
		CP-H18	TM6, TM5	TM2, TM1	-16.48 ± 0.35
CCR2-CCR5	AF2	AF-1	TM6, TM7 (E), TM5 (I)	Identical	69.24 ± 0.30
		**AF-2**	**TM5, TM4, TM3 (I)**	**Identical**	**-121.76 ± 0.14**
		AF-4	TM4	Identical	-3.90 ± 0.23
	Docking	CP-0	TM5, TM6	Identical	1.18 ± 0.23
		**CP-11**	**TM2, TM1, TM3 (E), TM4 (I)**	**TM7, TM1, TM6**	**-143.84 ± 0.24**
		CP-20	TM2, TM1 (I), TM3 (E)	TM6, TM5	-42.50 ± 0.34
CCR2-Iso_2TM	AF2	AF-1	TM6, TM7, TM5 (I)	TM1, TM2 (I)	-40.89 ± 0.19
		AF-5	TM5, TM4, TM3 (I)	TM1	-57.03 ± 0.11
	Docking	CP-0	TM2, TM1, TM3 (E), TM4 (I)	TM1, TM2	-123.96 ± 0.19
		CP-10	TM5, TM6	TM1, TM2	-74.12 ± 0.15
		**CP-13**	**TM2, TM1, TM3 (E), TM4 (I)**	**TM1, TM2**	**-149.04 ± 0.35**
		**CP-18**	**TM7, TM6, TM1**	**TM1, TM2**	**-103.16 ± 0.22**

Table S5. Summary of dimerization interfaces and BFE calculations of the complex structure models, for CCR7-related complexes

Complex	Model source	Model	Interface composition		Binding free energy (kcal/mol)
			CCR7	Interacting partner	
**CCR7-CCR7**	AF2	AF-1	TM1, TM2 (E)	Identical	-59.31 ± 0.20
		**AF-2**	**TM7, TM1**	**Identical**	**-118.45 ± 0.21**
		AF-3	TM5, TM3 (I), TM6 (E)	Identical	-55.93 ± 0.12
		AF-4	TM6, TM5 (I), TM7 (E)	Identical	13.19 ± 0.28
**CCR7-CXCR4**	AF2	AF-1	TM4, TM5	TM5, TM4	-89.33 ± 0.19
		**AF-2**	**TM7, TM6**	**TM6, TM7**	**-127.24 ± 0.26**
		AF-3	TM1, TM7(I)	TM5, TM6 (E)	-72.34 ± 0.22
		**AF-4**	**TM1, TM7 (I)**	**TM4, TM5**	**-119.23 ± 0.36**
		AF-5	TM1, TM7 (I)	TM6, TM7, TM5 (I)	-42.68 ± 0.24
	Docking	**CP-19**	**TM5, TM4, TM3 (I)**	**Identical**	**-117.10 ± 0.13**
		CP-H11	TM7, TM1, TM6 (M)	TM2, TM1, TM3 (E)	-90.25 ± 0.35
**CCR7-Iso_5TM**	AF2	AF-1	TM1, TM7 (I), TM2 (E)	TM1, TM2 (E)	-135.13 ± 0.24
		AF-2	TM2, TM4, TM3 (E)	TM4, TM3 (E), TM1(I)	-83.69 ± 0.20
		AF-4	TM6, TM7 (I), TM1 (I)	TM1, TM2 (I), TM5 (I)	-75.71 ± 0.29
	Docking	CP-11	TM1, TM2, TM7	TM3, TM2, TM1	-85.30 ± 0.23
		CP-20	TM5, TM4, TM3(E)	TM5, TM4, TM1, TM2	-127.05 ± 0.19
		**CP-H10**	**TM5, TM4, TM3(E)**	**TM5, TM4, TM1, TM2**	**-140.47 ± 0.22**
		**CP-H18**	**TM1, TM7, TM2**	**TM2, TM1, TM3**	**-152.19 ± 0.29**
